# circLOC375190 promotes autophagy through modulation of the mTORC1/TFEB axis in acute ischemic stroke-induced neurological injury

**DOI:** 10.1016/j.clinsp.2025.100581

**Published:** 2025-01-29

**Authors:** Qie Liu, Lu Zhang, Xin Xu

**Affiliations:** Department of Neurology, Daqing Oilfield General Hospital, Daqing City, Heilongjiang Province, China

**Keywords:** circLOC375190, mTORC1/TFEB pathway, Acute ischemic stroke, Autophagy, Neurological injury

## Abstract

**Objective:**

The authors explored differentially expressed circRNAs in Acute Ischemic Stroke (AIS) and revealed the role and potential downstream molecular mechanisms of circLOC375190.

**Methods:**

circLOC375190 expression was modulated by lentiviral injection in the brain of transient Middle Cerebral Artery Occlusion (tMCAO) mice. Neurological dysfunction was assessed, as well as infarction size, histopathological changes, and neuronal apoptosis in tMCAO mice. An in vitro Oxygen-Glucose Deprivation/Reoxygenation (OGD/R) PC-12 cell model was established. PC-12 cells were transfected and evaluated for viability, cytotoxicity, apoptosis, and autophagy. Inflammatory factors in mouse brain tissues and PC-12 cells were examined via enzyme-linked immunosorbent assay, and related genes were measured via real-time reverse transcriptase-polymerase chain reaction and Western blot. The ring structure of circLOC375190 was assessed by actinomycin-D and RNase-R assays. circRNA targeting to downstream factors was assessed by Fluorescence in situ hybridization assay, dual luciferase reporter assay, and RNA immunoprecipitation assay.

**Results:**

circLOC375190 level was increased in tMCAO mice. Knocking down circLOC375190 reduced infarct size, attenuated cerebral pathological injury and neuronal apoptosis, and inhibited inflammatory damage and autophagy in tMCAO mice. circLOC375190 knockdown enhanced neuronal viability and reduced cytotoxicity, apoptosis, and autophagy in OGD/R-treated PC12 cells. Mechanistically, circLOC375190 acted as a sponge for miR-93-5p to upregulate MAP kinase interacting serine/threonine kinase 2 expression and activate the mechanistic target of rapamycin complex 1/transcription factor EB pathway.

**Conclusion:**

circLOC375190 exacerbates tMCAO-mediated neurological injury by regulating neuronal autophagy.

## Highlights

• circLOC375190 reduction ameliorates neuronal autophagy and apoptosis in tMCAO mice.
• circLOC375190 knockdown improves OGD/R-mediated neuronal autophagy and apoptosis.
• circLOC375190 affects OGD/R-mediated neuronal apoptosis and autophagy by adsorbing miR-93-5p.
• circLOC375190 affects OGD/R-mediated neuronal apoptosis and autophagy by regulating the miR-93-5p/MKNK2 axis.
• circLOC375190 affects AIS autophagy by regulating the mTORC1/TFEB axis.

## Introduction

Acute Ischemic Stroke (AIS) is defined as sudden neurologic dysfunction caused by focal brain ischemia.^1^ Cerebral infarction stands as the foremost lesion resulting from AIS. Insufficient blood flow to brain tissue initially leads to a reversible decline in tissue functionality and eventually results in an infarction, characterized by the loss of neurons and supportive structures.^2^ Strokes can cause sudden numbness or weakness in arms or legs, facial droop, mental confusion, and vision loss.^3^ Advanced stroke management aims to perform revascularization and limit secondary neuronal injury. Intravenous thrombolysis and endovascular thrombectomy are now available.^1^^,^^4^

A significant amount of research has been conducted on identifying biomarkers that can be used to diagnose and treat patients with AIS.^5^, ^6^, ^7^ CircRNAs are a recently discovered class of non-coding RNAs that are generated by cyclization.^8^ Through miRNA/mRNA axis, CircRNAs can control nerves, blood vessels, and the immune system, thereby affecting the neurovascular unit affected by ischemia/reperfusion.^9^ A novel circRNA, circLOC375190 was speculated to regulate AIS-induced neurological injury through the miR-93-5p/MAP Kinase Interacting serine/threonine Kinase 2 (MKNK2) axis, which was validated in the present study. A neuroprotective role of miR-93-5p has been validated in the treatment of intracranial hemorrhage in neurological diseases.^10^ As a serine/threonine kinase, MKNK2 phosphorylates serine or threonine residues on its substrates. It coordinates cellular signaling, regulates inflammatory chemokine production, and regulates cell proliferation.^11^ However, the specific mechanism of circLOC375190/miR-93-5p/MKNK2 axis is still insufficiently explored.

Therefore, to provide a theoretical foundation for the treatment of AIS, the authors explored the specific mechanism of circLOC375190 by regulating the miR-93-5p/MKNK2 axis.

## Materials and methods

### Bioinformatics analysis

Open-access circRNA expression profiles and corresponding tissue information were obtained from the NCBI Gene Expression Omnibus (GEO database) project GSE133768. The authors extracted circRNA expression profiles from the GSE133768 project for 3 samples of healthy subjects and 3 samples of AIS patients with the annotation platform GPL21825. Differential genes in the GSE133768 database were analyzed via the bioinformatics website circmine (http://www.biomedical-web.com/circmine/home). Genes with Log2 Fold change > 1 and *p* < 0.05 were labeled as differential genes.

### Animals

Forty adult male C57BL/6J mice (25 ± 3 g, 8‒10-weeks-old) were obtained from Hunan SJA Laboratory Animal Co., Ltd. (Changsha, China). The mice were housed in a constant temperature and humidity environment with a 12-hour light-dark cycle and had free access to drinking water and basal chow. All animal experiments were approved by the Animal Care and Use Committee of Daqing Oilfield General Hospital. This study followed ARRVIE guidelines.

### Modeling of transient middle cerebral artery occlusion (tMCAO)

tMCAO was performed as described previously.^12^ The mice underwent anesthesia through 3 % isoflurane induction. The external right carotid artery was exposed, followed by the insertion of a silicone rubber-coated 6‒0 nylon filament (Doccol) along the internal carotid artery, extending 9‒10 mm to the carotid bifurcation and the middle cerebral artery. An hour subsequent to the blockage, the filament was extracted to reinstate blood circulation in the middle cerebral artery. Throughout the process, a noninvasive tail cuff (BP-2010, Softron Beijing Biotechnology) was employed to track systolic blood pressure. The body temperature of mice was kept at 37.0° ± 0.5 °C using a temperature-regulated heating pad throughout the surgical and recovery phases. In the group undergoing sham operations, mice were not subjected to nylon wire insertion. Rapamycin (4 mg/kg/day) was administered intraperitoneally for two days one week prior to surgery to block the mechanistic Target of Rapamycin Complex-1 (mTORC1) pathway.

### Adenoviral vector injection

Adenoviral shRNA vectors targeting circLOC375190 (OBio) were injected into the mouse ventricles three weeks before tMCAO surgery. For intracerebroventricular microinjection of AAV, neuron-specific control shRNA-GFP AAV and circLOC375190 shRNA-GFP AAV (4 × 10^10^ viral genomes/mouse in a volume of 1 μL) were infused into the left ventricle at 0.1 μL/min. A 10-minute break was required for diffusion. After 3 weeks of microinjection, the mice were divided into the following 4 groups: Sham group, tMCAO group, tMCAO+GFP-AAV group, and tMCAO+sh-circLOC375190-AAV group.

### Neurological scoring

The Zea-Longa experiment^13^ was performed after 6 h of reperfusion. 0: Completely normal behavior without any signs of neurological deficits; 1-point: Mild neurological deficits accompanied by impaired pulling ability in the left forelimbs; 2-points: Moderate neurological deficits, marked by the inability to move in a straight and forward direction due to continuous lateral body turns; 3-points: Severe neurological deficits, marked by the incapacity to move in a straight and forward direction owing to continuous lateral body turns; 4-points: Severe neurologic deficit, marked by the inability to stand and fall to the left when standing; 5-points: Death.

### 2,3,5-triphenyl tetrazolium chloride (TTC) staining

After neurological deficit scoring, the mice were anesthetized, the skulls were stripped, and the brains were rapidly removed. Fresh brain tissues from half of the mice in each group were taken for TTC staining, and the remaining brain tissues were fixed with 4 % paraformaldehyde or stored at −80 °C.

For TTC staining, 1-mm-thick coronal sections were serially sectioned and incubated in 2 % TTC solution for 30 min at 37 °C. Brain sections were further fixed with 4 % paraformaldehyde for 24 h, digitally scanned onto a computer, and analyzed using ImageJ software.Infarctvolume=infarctarea/wholebrainarea×100░%.

### Nissl staining and hematoxylin and eosin (HE) staining

Brain tissues were fixed with 4 % paraformaldehyde, paraffin-embedded, and cut into serial coronal sections using a cryostat. Then, the samples were routinely dewaxed, dehydrated, and stained with Nissl and HE solutions, respectively. Finally, the sections were sealed with neutral resin and observed by a Nikon Eclipse C1 microscope.

### Immunofluorescence staining

The sections underwent a 15-min incubation with 0.3 % Triton X-100 in Phosphate Buffered Saline (PBS) and were then treated with 10 % normal goat serum (ZSGB-BIO, ZLI-0956) in 0.3 % Triton X-100 for an hour at ambient temperature. Subsequently, the samples were subjected to an overnight incubation at 4 °C with the primary antibody and AlexaFluor-594-linked goat anti-mouse Immunoglobulin G (IgG) (Invitrogen, A-11,005) for one hour. The sections were placed on glass slides for microscopic imaging (Carl Zeiss, LSM700).

### Real-time reverse transcriptase-polymerase chain reaction (RT-qPCR)

RNA was extracted from brain tissues and cells using TRIzol reagent (Invitrogen, 15,596,026). Mature miRNAs were processed using a HiScript Q Select RT SuperMix for qPCR Kit (Vazyme, R133-01), followed by RNA reversal with stem-loop RT primers (RiboBio) and quantification via AceQ qPCR SYBR Green Master Mix (Vazyme, R141-02). U6 served as the internal standard. The reverse transcription of CircRNA and mRNA was performed with the HiScript Q RT SuperMix for qPCR Kit (Vazyme, R123-01), followed by quantification using SYBR Green Real-time PCR Master Mix (Vazyme, R141-02). Glyceraldehyde-3-phosphate dehydrogenase (GAPDH) served as the internal benchmark. Primer sequences are shown in Table 1.

**Table 1:** Primers used in RT-qPCR.

Genes	Primer sequences (5′–3′)
circFOXO3	Forward: 5′-GGACCCCAATTTTCTGAAGGC-3′
	Reverse: 5′-CAGCATCTCTTTTCTTCTTGTGT-3′
miR-93–5p	Forward: 5′-CAAAGTGCTGTTCGTGC-3′
	Reverse: 5′-TGGTGTCGTGGAGTCG-3′
MKNK2	Forward: 5′-ATCTATGACAAGCGCTGCGA-3′
	Reverse: 5′-CAGTCCTTGTCGGGGAACTC-3′
U6	Forward: 5′-CTCGCTTCGGCAGCACA-3′
	Reverse: 5′-AACGCTTCACGAATTTGCGT-3′
GAPDH	Forward: 5′-TCTGCTGTCTGTTACCTTGTCC-3′
	Reverse: 5′-TCTGCTGTCTGTTACCTTGTCC-3′

Note: GAPDH, Glyceraldehyde-3-Phosphate Dehydrogenase; MKNK2, MAP Kinase interacting serine/threonine Kinase 2.

### Western blot

Proteins were extracted from brain tissues and cells using radioimmunoprecipitation assay lysis buffer (P0013B, Beyotime, Nanjing, China). Bicinchoninic acid protein assay reagent (Thermo Fisher Scientific) was taken to measure protein concentration. Proteins were separated on Sodium Dodecyl Sulphate (SDS) polyacrylamide gels (10 % and 12 %) and electrophoretically transferred to polyvinylidene difluoride membranes (Bio-Rad). The membranes were subsequently incubated with primary antibodies overnight at 4 °C and horseradish peroxidase-conjugated goat anti-mouse IgG secondary antibody (7076P2, Cell Signaling Technology [CST]). Immunoreactivity was detected using an enhanced chemiluminescence detection system (Thermo Fisher Scientific). Primary antibody information was as follows: Autophagy-related Gene 5 (ATG5) (10,181-2-AP, Proteintech), p62 (5114, CST), Transcription Factor EB (TFEB) (4240, CST), p-S6 kinase (S6 K) (9205, CST), S6 K (2708, CST), p-eIF4E-Binding Protein (4EBP1) (2855, CST), and 4EBP1 (9644, CST).

### Cell culture

PC-12 cells (National Collection of Authenticated Cell Cultures, Shanghai, China) were cultured in Dulbecco's modified Eagle's medium (DMEM, Gibco, Invitrogen, USA) supplemented with 10 % Fetal Bovine Serum (FBS) (Gibco) and 1 % penicillin and streptomycin (Thermo Fisher Scientific). Cells were maintained at 37 °C in a fully humidified 5 % CO_2_ incubator (3111, Thermo Fisher Scientific). To inhibit the mTORC1 pathway, PC-12 cells were treated with Rapamycin (25 μM) for 72 h

### Actinomycin D and RNase R experiments

PC-12 cells underwent inoculation in six-well plates, with each well containing 5 × 10^5^ cells. After a day, cells were exposed to 2 μg/mL of actinomycin D (Sigma) and gathered at specified intervals. The stability of RNA was examined through RT-qPCR. RNA from PC-12 cells (10 μg) was processed with RNase R (3 U/g, Epicenter) and subsequently incubated at 37 °C for half an hour. The presence of CircRNA and linear RNA was identified through RT-qPCR analysis.

### Northern blotting

To conduct Northern blotting, RNA treated with Total RNA and RNase R was utilized, employing the NorthernMax® Kit (Invitrogen, Life Technologies). The specimens were processed on 1 % formaldehyde-polyacrylamide-urea gels, moved to Hybond N^+^ membranes with a positive charge (Amersham), and subsequently underwent cross-linking under UV light. Membranes were hybridized with either a 3′-digoxigenin-tagged circLOC375190 probe or a GAPDH probe (Axl-Bio, Guangzhou, China) at 50 °C throughout the night.

### Fluorescent in situ hybridization (FISH) assay

Neurons were fixed with 4 % paraformaldehyde and incubated with 0.25 % Triton X-100 in PBS for a duration of 15 min. The specimens were incubated in a hybridization solution (50 % formamide, 10 mm Tris–HCl, pH 8.0, 200 μg/mL yeast tRNA, 600 mm NaCl, 1 × Denhardt's solution, 0.1 mm ethylenediamine tetraacetic acid, 25 % SDS, and 10 % dextran sulfate) for an hour, followed by an overnight incubation at 37 °C with either a biotin-tagged circLOC375190 probe (50 nm, Invitrogen) or a digoxigenin-tagged miR-93–5p probe (25 nm, Invitrogen). Following three washes with TBS, the TSA Cy5 kit (PerkinElmer, NEL745001KT) was used to intensify the signals for a duration of 10 min. Subsequently, coverslips were affixed using Prolong Gold anti-fade reagent infused with 4′−6-diamidino-2-phenylindole and captured under a microscope (Carl Zeiss, LSM700).

### Cell transfection

The siRNA or pcDNA 3.1 overexpression vectors targeting circLOC375190 and MKNK2, miR-93-5p mimic/inhibitor, and their negative controls were purchased from Guangzhou RuiBo Bio. The above reagents were transfected into PC-12 cells using Lipofectamine 3000 (Invitrogen). After 48 h of transfection, the transfection efficiency was assessed by RT-qPCR or western blot.

### Oxygen-glucose deprivation/reoxygenation (OGD/R) treatment

PC-12 cells were cultured with glucose-free deoxygenated DMEM (Invitrogen, 11,966–025) in an incubator with premixed gas (95 % N_2_ and 5 % CO_2_) for 4 h Normal DMEM containing 10 % FBS was then given, and cells were placed in an incubator (95 % air and 5 % CO_2_) for 24 h Cells maintained in DMEM and 10 % FBS under normal conditions were used as controls.

### 3-(4,5-dimethylthiazol-2-yl)-2,5-diphenyltetrazolium bromide (MTT) assay

PC-12 cells were inoculated into 96-well plates, and then 20 μL of MTT (5 mg/mL, Sigma-Aldrich) was added. After 4 h of incubation at 37 °C, 150 μL of dimethyl sulfoxide (Sigma-Aldrich) was introduced into each well. Finally, absorbance was detected at 570 nm using a microplate reader (Bio-Tek Instruments, MA, USA).

The absorbance was measured using the Pierce Lactate Dehydrogenase (LDH) Cytotoxicity Assay Kit (Thermo Scientific) to assess cytotoxicity by quantitatively evaluating LDH release from the culture medium according to the kit protocol.

### Flow cytometry

Apoptosis of PC-12 cells was detected using the Annexin V/Fluorescein isothiocyanate (FITC) Apoptosis Detection Kit (Southern Biotech, AL, USA). Cells were washed with PBS (Invitrogen) and resuspended in a binding buffer. After staining with 5 μL AnnexinV-FITC/propidium iodide (Bender Med System) for 15 min, apoptosis was analyzed by FACSan flow cytometry (BD Bioscience, Heidelberg, Germany).

### Enzyme-linked immunosorbent assay (ELISA)

Levels of Tumor Necrosis Factor (TNF)-α, Interleukin (IL)-1β, and IL-6 in brain tissue and cells were measured using commercial kits (Abcam).

### Monodansylcadaverine (MDC) staining

PC-12 cells were seeded in 6-well plates, followed by a PBS wash and a 20-min incubation period at 37 °C with 50 μM MDC. Cells were fixed with 4 % paraformaldehyde for 15 min and examined using MetaMorph software and fluorescent microscopy (Olympus, BX51).^14^

### Dual luciferase reporter assay

Using human genomic DNA, PCR was used to amplify the circLOC375190 and MKNK2 3′-UTR harboring miR-93-5p binding sites. This DNA fragment, designated WT-circLOC375190/MKNK2, was cloned into the pmiR-RB-REPORT vector (RiboBio) at the XhoI and NotI sites. To create a mutant luciferase reporter vector, the miR-93-5p binding site in the circLOC375190 and MKNK2 3′-UTR was mutated. HEK293T cells were cotransfected with the luciferase reporter vectors and miR-93-5p mimic or mimic NC using Lipofectamine 3000 (Invitrogen). Luciferase activity was evaluated 24 h after transfection (Promega, E2920).

### RNA immunoprecipitation (RIP) assay

The RIP solution containing magnetic beads containing either mouse IgG or human anti-Ago2 antibody was added to cell lysates. Proteinase K was used to extract the complexes, and the immunoprecipitated RNA was subsequently separated. A spectrophotometer (NanoDrop, Thermo Scientific) was utilized to determine the concentration of RNA and evaluate its quality. RT-qPCR was employed to evaluate pure RNA and show the existence of binding targets.

### Data analysis

Data were expressed as mean ± Standard Deviation (SD). All experiments were performed with at least three biological replicates. Statistical analysis was performed using GraphPad Prism 9.0. Two-group comparisons were performed using the Student's *t*-test, and one-way analysis of variance (ANOVA) was used for multiple-group comparisons; *p* < 0.05 was considered statistically different.

## Results

### circLOC375190 is abnormally highly expressed in AIS

**Fig. 1:**
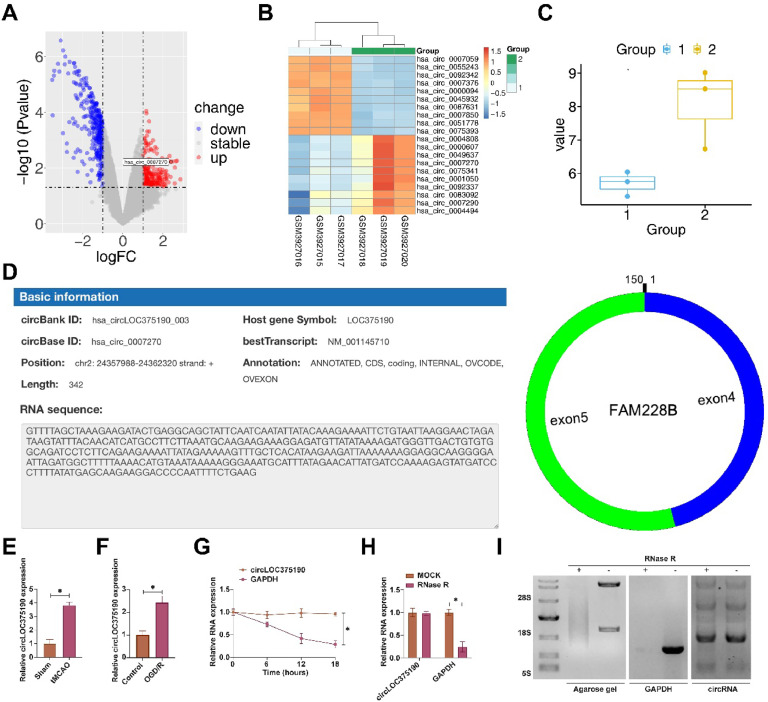
circLOC375190 is abnormally highly expressed in AIS. (A) Volcano plot of differentially expressed circRNAs in plasma from three healthy subjects and three AIS patients; (B) Heat map analysis of 10 up-regulated and down-regulated circRNAs; (C) ANOVA detection of circLOC375190 expression pattern in plasma from three healthy subjects and three AIS patients; (D) Starbase to query genetic information of circLOC375190; (E and F) RT-qPCR to detect circLOC375190 in brain tissues of tMCAO mice or OGD/R-treated PC-12 cells; (G‒I) Actinomycin D, RNase R, and Northern blotting to verify the circLOC375190 circular structure; data are expressed as mean ± SD (*n* = 3).

The authors analyzed differentially expressed circRNAs in plasma from three healthy subjects and three AIS patients from the bioinformatics website (http://www.biomedical-web.com). Among the top 10 abnormally highly expressed circRNAs, hsa_circ_0007270 has both murine and human sequences, therefore, it was identified as the circRNA of interest and named circLOC375190 (with log2 fold change of 2.3931) (Fig. 1A‒C). By circbase query, hsa_circ_0007270 was located on exons 4 and 5 of the LOC375190 gene with a length of 342 bp (Fig. 1D). Subsequently, the authors examined circLOC375190 expression in tMCAO mice and OGD/R-treated PC-12 cells. Either tMCAO or OGD/R treatment promoted circLOC375190 expression (Fig. 1E‒F). Subsequently, the authors explored the ring structure of circLOC375190. Actinomycin D assay showed that circLOC375190 exhibited a longer half-life compared to linear GAPDH mRNA (Fig. 1G). Northern blotting and RNase R experiments showed that RNase R reduced linear GAPDH mRNA levels but had no effect on hsa_circ_0007270 (Fig. 1H‒I).

### circLOC375190 reduction ameliorates neuronal autophagy and apoptosis in tMCAO mice

**Fig. 2:**
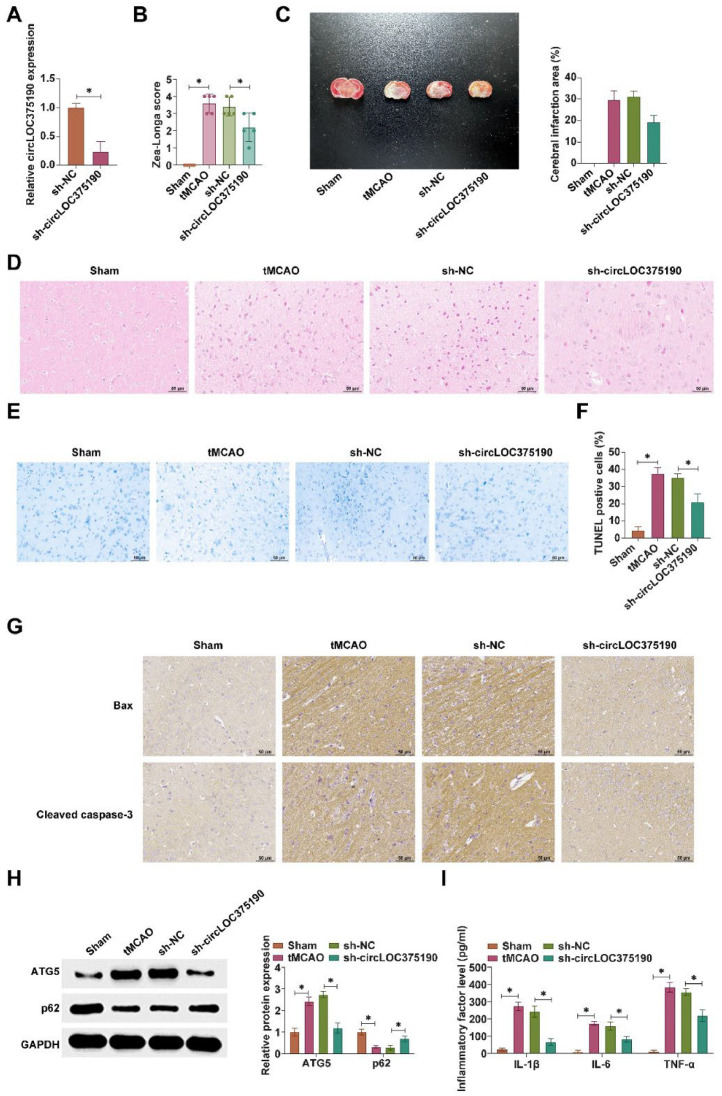
Knockdown of circLOC375190 improves neuronal autophagy and apoptosis in tMCAO mice. The shRNA adenoviral vector targeting circLOC375190 was injected into the mouse ventricles. (A) RT-qPCR to detect the expression of circLOC375190; (B) Neurological function scores in each group of mice; (C) TTC staining to detect the area of cerebral infarction in mice; (D‒E) HE staining and Nissl staining of the brain tissues of mice in each group; (F) IHC staining to assess the positive rate of Bax and cleaved caspase-3 in brain tissue; (G) Western blot to detect the expression of ATG5 and P62 in brain tissue; (H) ELISA to detect the levels of IL-1β, IL-6 and TNF-α in brain tissue; data are expressed as mean ± SD (*n* = 5).

Subsequently, the authors injected shRNA adenoviral vectors targeting circLOC375190 into the mouse ventricles. RT-qPCR confirmed the successful knockdown of circLOC375190 in the ventricles of tMCAO mice (Fig. 2A). Neurological function scoring demonstrated significant improvement of neurological deficits in tMCAO mice after reducing circLOC375190 (Fig. 2B). Subsequently, infarct area was assessed by TTC staining. Knocking down circLOC375190 reduced the cerebral infarct area in tMCAO mice (Fig. 2C). In addition, the degree of brain tissue damage in mice was further evaluated by HE-staining and Nissl staining. circLOC375190 knockdown effectively ameliorated brain tissue injury in tMCAO mice increased the number of Nissl bodies and decreased the rate of apoptosis in brain tissues (Fig. 2D‒E). In addition, apoptosis-related proteins were evaluated using IHC staining. circLOC375190 knockdown decreased the positive rate of Bax and cleaved caspase-3 in brain tissues (Fig. 2F). Western blot demonstrated that circLOC375190 knockdown decreased ATG5 expression and promoted p62 expression (Fig. 2G). ELISA experiments confirmed that the knockdown of circLOC375190 decreased IL-1β, IL-6, and TNF-α levels in brain tissues of tMCAO mice (Fig. 2H).

### circLOC375190 knockdown improves OGD/R-mediated neuronal autophagy and apoptosis

**Fig. 3:**
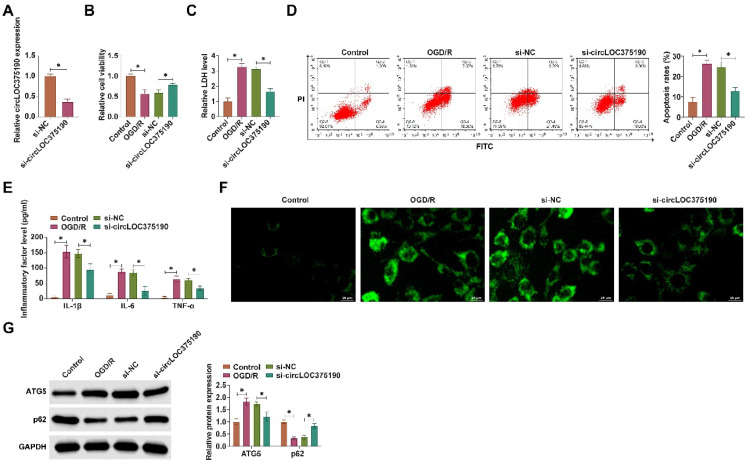
Knockdown of circLOC375190 ameliorates OGD/R-mediated neuronal autophagy and apoptosis. siRNA targeting circLOC375190 was transfected into OGD/R-treated PC-12 cells. (A) RT-qPCR to detect circLOC375190 expression; (B) MTT assay to detect cell viability; (C) Commercial kit to detect LDH release; (D) Flow cytometry to detect apoptosis; (E) ELISA to assess the levels of inflammatory factors IL-1β, IL-6 and TNF-α levels; (F) MDC staining to detect the formation of cellular autophagic vesicles; (G) Western blot to detect the expression of ATG5 and P62 in the cells; data are expressed as mean ± SD (*n* = 3).

Subsequently, PC-12 cells were subjected to OGD/R treatment to mimic AIS in vitro and transfected with siRNA targeting circLOC375190. RT-qPCR confirmed the successful knockdown of circLOC375190 upon si-circLOC375190 transfection (Fig. 3A). Subsequently, cell viability was assessed by MTT assay, and it was found that circLOC375190 knockdown effectively restored PC-12 cell viability (Fig. 3B) and reduced the release of LDH, thereby reducing cytotoxicity (Fig. 3C). Flow cytometry demonstrated that downregulating circLOC375190 reduced the apoptosis rate of PC-12 cells (Fig. 3D). ELISA results presented that downregulating circLOC375190 reduced IL-1β, IL-6, and TNF-α levels in cell supernatants (Fig. 3E). To assess the effect of circLOC375190 on OGD/R-mediated neuronal autophagy, the formation of cellular autophagic vesicles was first evaluated by MDC staining. Suppressing circLOC375190 significantly reduced the number of autophagic vesicles (Fig. 3F). Western blot confirmed that downregulating circLOC375190 decreased ATG5 and forced p62 expression (Fig. 3G).

### circLOC375190 competitively binds miR-93-5p

**Fig. 4:**
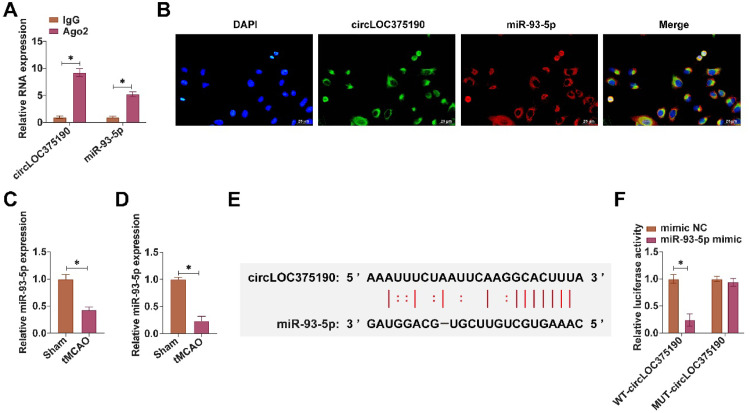
circLOC375190 competitively binds miR-93-5p. (A) RIP assay to detect the binding relationship between miR-93-5p and circLOC375190; (B) FISH assay to detect the subcellular localization of miR-93-5p with circLOC375190; (C and D) RT-qPCR to detect miR-93-5p expression in tMCAO mice and OGD/R-treated PC-12 cells; (E) Predicted binding sites of miR-93-5p with circLOC375190; (F) Dual luciferase reporter assay to detect the targeting relationship of miR-93-5p with circLOC375190; data are expressed as mean ± SD (*n* = 3).

Subsequently, the authors explored the downstream miRNAs that bind to circLOC375190. The authors selected miRNAs with predicted binding sites to circLOC375190 from the bioinformatics website starbase. RIP experiments revealed that miR-93-5p was highly enriched with circLOC375190 in Ago2 magnetic beads (Fig. 4A). Subsequently, their subcellular localization was analyzed by FISH assay. Both miR-93-5p and circLOC375190 were localized in the cytoplasm of PC-12 cells (Fig. 4B). miR-93-5p was lowly expressed in both tMCAO mice and OGD/R-treated PC-12 cells (Fig. 4C‒D). circLOC375190 shared binding sites with miR-93-5p (Fig. 4E). Dual-luciferase reporter assays showed that WT-circLOC375190 reduced the luciferase activity of cells after cotransfection with miR-93-5p mimic (Fig. 4F).

### circLOC375190 affects OGD/R-mediated neuronal apoptosis and autophagy by adsorbing miR-93-5p

**Fig. 5:**
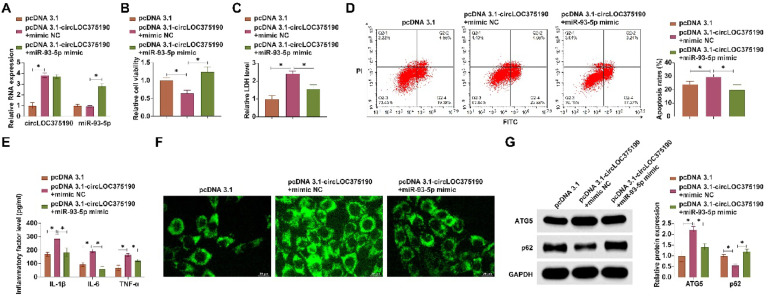
circLOC375190 affects OGD/R-mediated neuronal apoptosis and autophagy by adsorbing miR-93–5p. pcDNA 3.1-circLOC375190 and miR-93-5p mimic were co-transfected into OGD/R-treated PC-12 cells. (A) RT-qPCR to detect circLOC375190 expression; (B) MTT assay to detect cell viability; (C) Commercial kit to detect LDH release; (D) Flow cytometry to detect apoptosis; (E) ELISA to assess the levels of inflammatory factors IL-1β, IL-6 and TNF-α levels; (F) MDC staining to detect the formation of cellular autophagic vesicles; (G) Western blot to detect the expression of ATG5 and P62 in the cells; data are expressed as mean ± SD (*n* = 3).

The authors cotransfected pcDNA 3.1-circLOC375190 and miR-93-5p mimic into OGD/R-treated PC-12 cells. RT-qPCR confirmed that pcDNA 3.1-circLOC375190 increased circLOC375190 expression and did not affect miR-93-5p expression, while miR-93-5p mimic increased miR-93-5p levels (Fig. 5A). Functional rescue experiments showed that pcDNA 3.1-circLOC375190 further inhibited cell viability, promoted LDH release, increased apoptosis rate, promoted the production of inflammatory factors, increased the number of autophagic vesicles, promoted ATG5 expression, and inhibited p62 expression, but these effects were all rescued by miR-93-5p mimic (Fig. 5B‒G).

### MiR-93-5p targets and regulates MKNK2 expression

**Fig. 6:**
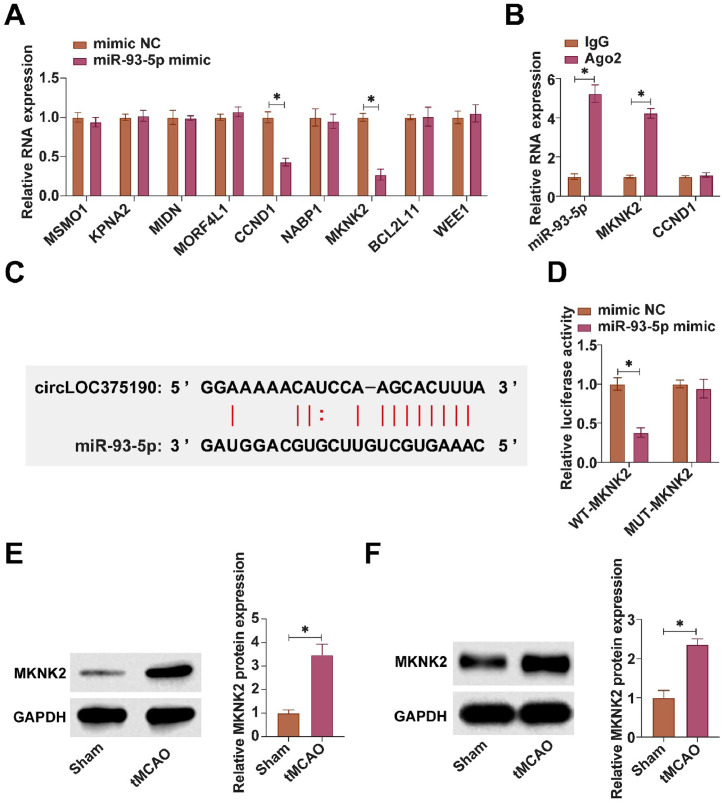
MiR-93-5p targets and regulates MKNK2 expression. (A) RT-qPCR to detect mRNAs negatively regulated by miR-93-5p; (B) RIP assay to detect the binding relationship between miR-93-5p and MKNK2; (C) Predicted binding sites of miR-93-5p and MKNK2; (D) Dual-luciferase reporter assay to detect the targeting relationship between miR-93-5p and MKNK2; (E and F) Western blot to detect MKNK2 expression in tMCAO mice and OGD/R-treated PC-12 cells; data are expressed as mean ± SD (*n* = 3).

Subsequently, the authors analyzed the downstream target genes of miR-93-5p. The top 10 downstream target genes of miR-93-5p with the most data support from AGO Clip-seq experiments were selected from bioinformatics websites. RT-qPCR showed that only MKNK2 and cyclin D1 were negatively regulated by miR-93-5p in PC-12 cells (Fig. 6A). MKNK2 and miR-93-5p were highly enriched in Ago2 magnetic beads as confirmed by RIP assay (Fig. 6B). Based on the predicted binding site, WT/MUT-MKNK2 was designed (Fig. 6C). Co-transfection of WT-MKNK2 and miR-93-5p mimic reduced luciferase activity in the dual reporter assay (Fig. 6D). High expression of MKNK2 was found in both tMCAO mice and OGD/R-treated cells (Fig. 6E‒F).

### circLOC375190 affects OGD/R-mediated neuronal apoptosis and autophagy by regulating the miR-93-5p/MKNK2 axis

**Fig. 7:**
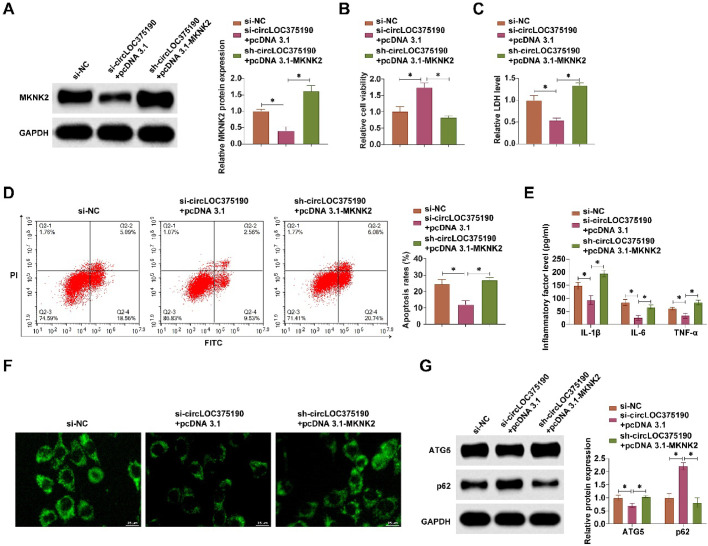
circLOC375190 affects OGD/R-mediated neuronal apoptosis and autophagy by regulating miR-93-5p/MKNK2 axis. si-circLOC375190 and pcDNA 3.1-MKNK2 were co-transfected into OGD/R-treated PC-12 cells. (A) Western blot to detect circLOC375190 expression; (B) MTT assay to detect cell viability; (C) Commercial kit to detect LDH release; (D) Flow cytometry to detect apoptosis; (E) ELISA to assess the levels of inflammatory factors IL-1β, IL-6 and TNF-α levels; (F) MDC staining to detect the formation of cellular autophagic vesicles; (G) Western blot to detect the expression of ATG5 and P62 in the cells; data are expressed as mean ± SD (*n* = 3).

The authors cotransfected si-circLOC375190 and pcDNA 3.1-MKNK2 into OGD/R-treated PC-12 cells. si-circLOC375190 decreased MKNK2 expression, but this effect was blocked by pcDNA 3.1-MKNK2 (Fig. 7A). Functional rescue experiments showed that si-circLOC375190 increased cell viability, reduced LDH release, decreased apoptosis, inhibited the production of inflammatory factors, decreased the number of autophagic vesicles, inhibited ATG5 expression, and promoted p62 expression, but all these effects were disrupted by pcDNA 3.1-MKNK2 (Fig. 7B‒G).

### circLOC375190 affects AIS autophagy by regulating the mTORC1/TFEB axis

**Fig. 8:**
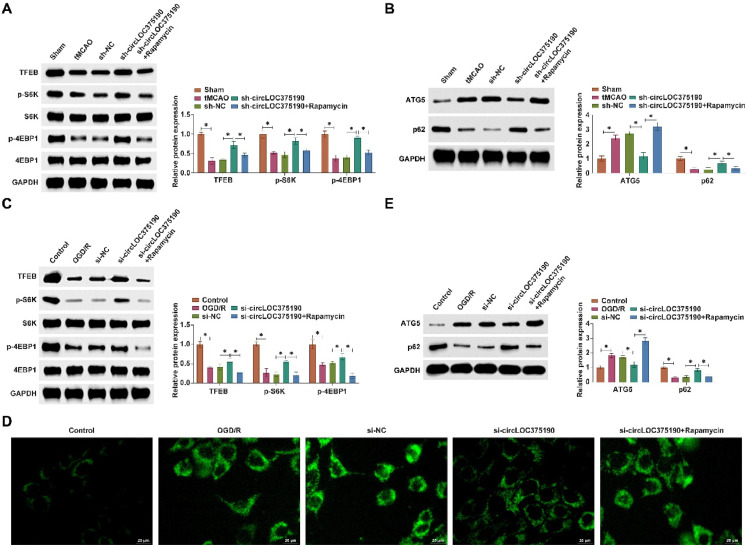
circLOC375190 affects AIS autophagy by modulating the mTORC1/TFEB axis. The mTORC1 pathway was blocked using a mTORC1 inhibitor (Rapamycin) in tMCAO mice or OGD/R-treated cells that knocked down circLOC375190. (A) Western blot to detect TFEB, p-S6 K, and p-4EBP1 in the brain tissues of each group of mice; (B) Western blot to detect ATG5, and P62 in each group of mouse rat brain tissues; (C) Western blot to detect the expression of TFEB, p-S6 K and p-4EBP1 in cells; (D) MDC staining to detect the formation of autophagic vesicles in cells; (E) Western blot to detect ATG5 and P62 in cells.

The authors used a mTORC1 inhibitor (Rapamycin) to block the mTORC1 pathway. Downregulating circLOC375190 effectively restored TFEB, p-S6K, and p-4EBP1 expression in the brain tissue of tMCAO mice, but this effect was blocked by Rapamycin (Fig. 8A). In addition, the inhibitory effects of knockdown of circLOC375190 on autophagy in tMCAO mice were all reversed by Rapamycin (Fig. 8B). circLOC375190 downregulation promoted TFEB, p-S6K, and p-4EBP1 levels in OGD/R-treated PC-12 cells, which was blocked by Rapamycin (Fig. 8C). The inhibitory effect of circLOC375190 silencing on autophagy was reversed by Rapamycin (Fig. 8D‒E).

## Discussion

It is a public health priority because strokes are associated with a high death and disability rate.^15^ In response to stroke, cells use autophagy as an adaptive mechanism to reduce stress by eliminating persistent or damaged organelles, protein clusters, and excess cellular elements through the autophagosome-lysosome degradation mechanism. Thus, autophagy is essential for maintaining cellular homeostasis and organism survival.^16^ The autophagy process in the brain is activated by ischemic stroke in neurons, glial cells, and brain microvascular cells.^17^ Despite ambiguous and inconsistent findings regarding the role of autophagy in the pathogenesis of IS, moderate activation of autophagy appears to be neuroprotective, whereas excessive activation of autophagy is detrimental.^18^ In the context of AIS, this study mainly assessed the regulatory action of circLOC375190 in neuronal autophagy and apoptosis and finally discovered that knocking down circLOC375190 exerted a neuroprotective role in tMCAO mice and OGD/R-treated neurons. circLOC375190 was initially detected to be abnormally high in plasma from AIS patients and determined as an interest of study due to its possible involvement in AIS. Because approximately 80 % of AIS occurs in the middle cerebral artery, a number of animal stroke models centered on this artery have been developed. Transient occlusions can be modeled with the tMCAO technique.^19^ The present study design is based on the mouse model of tMCAO, which revealed that suppression of circLOC375190 attenuated neurological deficit, reduced infarction, and improved brain injury. Moreover, circLOC375190 knockdown reduced apoptosis, autophagy, and inflammatory response. Cultured cells exposed to hypoxia and reoxygenation mimic the main features of ischemia-reperfusion, but can also adapt to treatments such as siRNA inhibition of certain genes and pharmacological activation or inhibition of signaling pathways, which is not possible in more complex settings.^20^ In vitro studies were prepared using OGD/R-treated neurons. It was measured that downregulating circLOC375190 restored viability and reduced cytotoxicity, apoptosis, release of inflammatory factors, and autophagy in OGD/R neurons. This study elucidated for the first time the protective action of silencing circLOC375190 in the setting of AIS. circLOC375190 was then investigated to competitively bind to miR-93-5p, a downregulated miRNA in tMCAO mice and OGD/R PC-12 cells. Functional assays were performed in OGD/R PC-12 cells, demonstrating that miR-93-5p overexpression reduced the detrimental influence of circLOC375190. ICH may be treated by targeting miR-93-5p as a therapeutic target.^10^ Further, in acute myocardial infarctions, miR-93-5p inhibits autophagy and inflammatory response, preventing cardiac injury.^21^ Furthermore, miR-93-5p overexpression inhibits the production of proinflammatory factors and restores HK-2 cell viability after hypoxia/reoxygenation stimulation.^22^ A study has shown that up-regulation of miR-93-5p suppresses autophagy in glaucoma-affected retinal ganglion cells.^23^

MKNK2 was a studied target of miR-93-5p in this study, which was highly expressed in both AIS models. The authors observed that elevating MKNK2 blocked circLOC375190 downregulation-induced protection against neuronal injury caused by OGD/R. In mice with trigeminal neuralgia, miR-223-3p attenuates neuropathic pain and reduces proinflammatory cytokines through its target, MKNK2.^24^ miR-93-5p in cardiomyocytes markedly reduces MKNK2 levels, resulting in lower phosphorylation of p38-MAPK pathways, subsequently mitigating oxidative stress damage in these cells.^25^ Silencing of MNK2 leads to diminished histopathological alterations in the lungs, evidenced by lower neutrophil levels and reduced IL-6 and TNF-α production in cases of acute lung injury.^26^

At last, the mTORC1/TFEB pathway was validated to be involved in circLOC375190 regulating autophagy. Specifically, mTORC1 inhibitor (Rapamycin) prevented the autophagy-inhibiting effect of circLOC375190 silencing. TFEB is a transcription factor that plays a key role in the control of lysosomal biogenesis and autophagy and is phosphorylated by the mechanistic target of mTORC1 in a manner that is distinct from the recruitment of substrates by mTORC1.^27^ mTORC1/TFEB signaling has been identified to play a role in autophagy in cardiomyocytes after myocardial ischemia-reperfusion injury.^28^^,^^29^

In conclusion, this study initially revealed that suppression of circLOC375190 reduces neuronal autophagy to rescue AIS through upregulating miR-93-5p to mediate the MKNK2-regulated mTORC1/TFEB pathway. This main finding provides a novel molecular target for developing a therapeutic target for AIS.

## Ethical statement

All animal experiments complied with the ARRIVE guidelines and performed in accordance with the National Institutes of Health Guide for the Care and Use of Laboratory Animals. The experiments were approved by the Institutional Animal Care and Use Committee of Daqing Oilfield General Hospital (n° 2020DQ0425).

## Availability of data and materials

The data that support the findings of this study are available from the corresponding author, upon reasonable request.

## Authors’ contributions

Conceptualization, Qie Liu and Lu Zhang; methodology, Lu Zhang and Xin Xu; formal analysis, Qie Liu; investigation, Lu Zhang; data curation, Xin Xu; writing-original draft preparation, Qie Liu and Lu Zhang; writing-review and editing, Qie Liu and Xin Xu; project administration, Qie Liu. All authors have read and agreed to the published version of the manuscript.

## Funding

Not applicable.

## Declaration of competing interest

The authors declare no conflicts of interest.
